# A Heat Vulnerability Index: Spatial Patterns of Exposure, Sensitivity and Adaptive Capacity for Santiago de Chile

**DOI:** 10.1371/journal.pone.0162464

**Published:** 2016-09-08

**Authors:** Luis Inostroza, Massimo Palme, Francisco de la Barrera

**Affiliations:** 1 Institute of Photogrammetry and Remote Sensing, Dresden University of Technology, D-01062, Dresden, Germany; 2 Universidad Autónoma de Chile, Temuco, Chile; 3 Escuela de Arquitectura, Universidad Católica del Norte, Angamos 610, Antofagasta, Chile; 4 Instituto Nacional de Eficiencia Energética y Energía Renovable, Iñaquito y Serrano, Quito, Ecuador; 5 Instituto de Geografía & Centro del Desarrollo Urbano Sustentable, Pontificia Universidad Católica de Chile, Vicuña Mackenna 4860, Macul, Santiago de Chile; Columbia University, UNITED STATES

## Abstract

Climate change will worsen the high levels of urban vulnerability in Latin American cities due to specific environmental stressors. Some impacts of climate change, such as high temperatures in urban environments, have not yet been addressed through adaptation strategies, which are based on poorly supported data. These impacts remain outside the scope of urban planning. New spatially explicit approaches that identify highly vulnerable urban areas and include specific adaptation requirements are needed in current urban planning practices to cope with heat hazards. In this paper, a heat vulnerability index is proposed for Santiago, Chile. The index was created using a GIS-based spatial information system and was constructed from spatially explicit indexes for exposure, sensitivity and adaptive capacity levels derived from remote sensing data and socio-economic information assessed via principal component analysis (PCA). The objective of this study is to determine the levels of heat vulnerability at local scales by providing insights into these indexes at the intra city scale. The results reveal a spatial pattern of heat vulnerability with strong variations among individual spatial indexes. While exposure and adaptive capacities depict a clear spatial pattern, sensitivity follows a complex spatial distribution. These conditions change when examining PCA results, showing that sensitivity is more robust than exposure and adaptive capacity. These indexes can be used both for urban planning purposes and for proposing specific policies and measures that can help minimize heat hazards in highly dynamic urban areas. The proposed methodology can be applied to other Latin American cities to support policy making.

## 1. Introduction

South American cities are expanding at impressive rates of more than 20 m^2^ per minute, concentrating more than 85% of the total population [1]. This expansion has generated a pronounced spatial pattern of social segregation where the low-income population tends to reside in poorly developed neighborhoods compared to high-income population [2]. Climate change (CC) will worsen this situation, as low-income population in new suburban neighborhoods tend to settle in environmentally phrone areas in high levels of urban informality [3] whose dwelling solutions are precarious to cope with increasing climate stressors. More detailed information on vulnerability at city and neighbourhood scales is needed for the development of specific CC adaptation strategies [4]. Spatial assessments of CC impacts at the urban level must evolve from simple measures based on physical (expected) events to the use of broader social indexes. Expert knowledge and views are frequently used in the development, aggregation and weighting of indexes, although a theoretical background on their development is often missing. Data availability always plays a crucial role in determining the selection of variables [5]. However, there is insufficient knowledge available to develop a better understanding of social vulnerabilities due to climate in the future [6]. The identification and prioritization of urban areas according to specific threats, development trends and local capacities is particularly pivotal [4]. Urban planning mechanisms must urgently incorporate adaptation strategies to cope with the scope and intensity of future CC impacts [7,8,9].

The aim of this study is to analyse heat vulnerability in Santiago, Chile. This city has a semi-arid Mediterranean climate with continental influence which implies high daily thermal oscillation and high temperatures in summer days. Santiago is a good representative of metropolitan areas in Latin-america characterized by a fast growing areas and the unequal distribution of positive environmental conditions [10]. A heat vulnerability index (HVI) is proposed. This is composed of spatially explicit indexes of exposure, sensitivity and adaptive capacity. The specific objectives of the HVI are 1) to explore patterns in the spatial distribution of exposure, sensitivity, adaptive capacity and heat vulnerability and 2) to identify spatial clusters of areas presenting high levels of heat vulnerability to explore their driving factors at census tract scale.

The impacts of heat waves are an emerging environmental and health concern [11], because the intensity and frequency of periods of extremely hot weather will increase, resulting in significant effects on human health [12, 13]. Even though until now heat has not been considered a relevant factor in assessing urban vulnerability in Chile [4] some recent studies have presented evidence of the existence of certain (localized) relationships between social vulnerability and CC impacts [14,15,16,17]. More research studies must develop urban heat maps and information on the climatic and socioeconomic relationships of heat-related health problems to better inform urban planning and policy making measures [9]. Urban environments are especially vulnerable to heat due to physical and social conditions that increase sensitivity. Concrete and building materials retain heat. Population density levels and green areas have been identified as main drivers of urban heat and health relationships [18,19,20]. However, higher population density levels and fewer green areas have not yet been directly related to socioeconomic categories: it is possible to find various relationships across different urban settings. Drivers of heat vulnerability are central to understanding the social construction of risk, how this may play out on in terms of environmental justice and ultimately the different societal impacts of extreme weather and climate events [15].

Heat vulnerability is linked to characteristics of individuals, buildings and urban structures. Heat stress becomes aggravated due to reduced levels of evapotranspiration and occurs in high-density industrial parks and in central zones, thus affecting public health [21]. Factors that influence heat vulnerability include the quality of housing and of the built environment, local urban geographies, resident lifestyles, income levels, employment trends, tenure patterns, social networks and self-perceptions of risk [22]. These factors influence an individual’s exposure and sensitivity to high temperatures as well as their capacities to anticipate, respond and adapt to conditions of heat stress. Many of these factors tend to overlap. For example, low-income population who lives in high-rise social housing buildings in city centres is likely to be more vulnerable to high temperatures [6,22]. Thus, a certain degree of correlation is to be expected. Heat vulnerability can be understood as the product of the effects of exposure, sensitivity and adaptive capacity [23]. Quantifying urban vulnerability to heat hazards through quantitative spatially explicit methods allows designing simple adaptation metrics, to identify location-specific measures at lower urban scales to assess the spatial configuration of vulnerability and its socioeconomic implications. Urban planning will play a key role in adapting the urban system to such trends [24].

## 2. Material and Methods

### 2.1. Study Area

**Fig 1:**
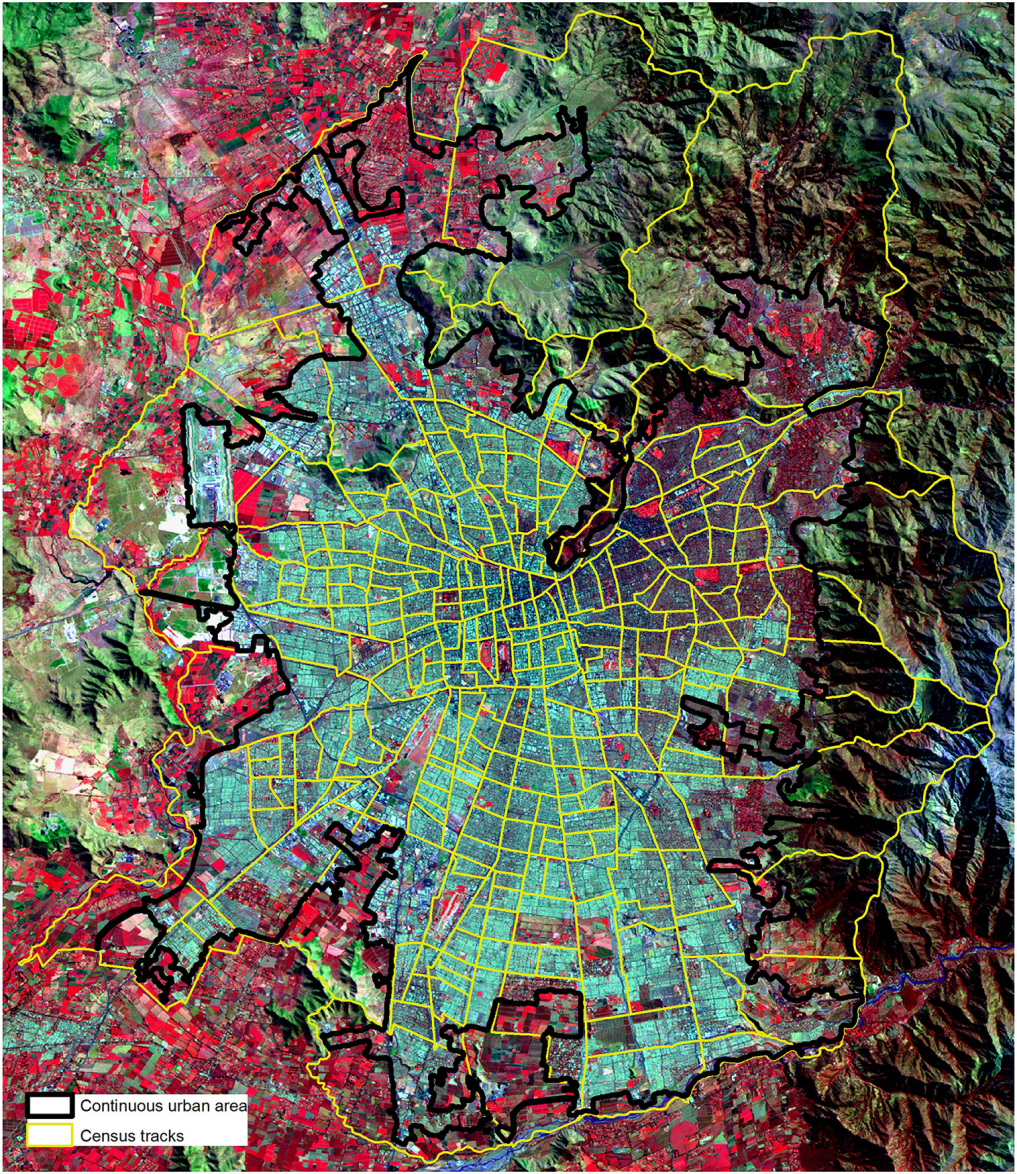
Continuous urban area and 277 census tracts used in the analysis. Background image: NASA Landsat Program, 2010, Landsat 5TM+ scene 20100319.141244/233_083_0/2, Bands 472, USGS, Sioux Falls, 03/19/2010.

Santiago (33.5° S, 70.6° W) is located in the valley of Central Chile, which is surrounded by two longitudinal ranges: the Andes and the Costal range (Fig 1). In addition to oceanic effects, these geographic attributes create a Mediterranean climate with long, dry summers and short rainy seasons during the winter characterized by 150 to 600 mm of precipitation. Air temperatures can reach 37 degrees Celsius or higher during the summer and are further amplified in vulnerable urban areas by urban heat island intensities that can approach 7 degrees Celsius [25,26,27]. Santiago is the capital of Chile and home to more than 43% of the Chilean population, which is estimated at 7.0 million [28]. In recent decades, the city has experienced considerable levels of urban expansion, adding more than 1,300 hectares/year (i.e., roughly 25 m^2^ per minute) to its continuous urban fabric at an average net population density of roughly 94 inhabitants per hectare [1]. However this high-density pattern is high variable, ranging from 40 to up to 166 inhabitants per hectare [29]. The city’s urban morphology is composed of contrasting land uses, population densities, housing types and vegetation, creating different thermal zones. The warmest areas generally lack green infrastructure and correspond with neighbourhoods dominated by low-income populations. In contrast, the coolest areas correspond with high-income neighbourhoods with better access to green infrastructure and lower density levels [14]. Thus, urban climatic conditions correlated to the socioeconomic characteristics of urban tissues [14].

The census, developed by the National Statistics Chilean Institute (INE), was used as the spatial unit of analysis. The study area covers 277 census tracts (Fig 1). The spatial assessment focused on the continuous urban area of Santiago, which corresponds to the sealed surfaces for the year 2010 determined through a spatial assessment of a Landsat image using a land cover driven approach and a buffer zone of 100 m [30, 1] (Table 1). This spatial data set was obtained from [1]. Only census tracts within this area were studied in the analysis. Census tracts located along the city’s periphery were in many cases very large or included important extensions of undeveloped land outside of the continuous urban area. To avoid generating biases in the calculations of pixel-based indexes, only the continuous urban area within the specific census tracts was used.

**Table 1:** Summary of data sources for the variables used in the analysis.

Index		Variable	Data description	Year	Data source
All		Continuous urban area	Built up surfaces and open spaces	2010	Landsat ETM+ / 25.10.2010
Exposure	1	Land Surface Temperature (LST)	Land surface temperature	2002	Landsat ETM+ / 21.03.2002
	2	Land Surface Temperature (LST)	Land surface temperature	2005	Landsat TM / 03.03.2005
	3	Land Surface Temperature (LST)	Land surface temperature	2009	Landsat TM / 16.03.2009
Sensitivity	1	Elderly population	Inhabitants per hectarea above 60 years old	2002	Instituto Nacional de Estadística de Chile (Censo de Población y Vivienda 2002)
	2	Very young population	Inhabitants per hectarea below 5 years old	2002	Instituto Nacional de Estadística de Chile (Censo de Población y Vivienda 2002)
	3	Disabled population	Inhabitants per hectarea handicapped	2002	Instituto Nacional de Estadística de Chile (Censo de Población y Vivienda 2002)
	4	Family structure	Inhabitants per hectarea single	2002	Instituto Nacional de Estadística de Chile (Censo de Población y Vivienda 2002)
	5	Education level	Inhabitants per hectarea with lower education	2002	Instituto Nacional de Estadística de Chile (Censo de Población y Vivienda 2002)
	6	Unemployment	Inhabitants per hectarea without a permanent employment	2002	Instituto Nacional de Estadística de Chile (Censo de Población y Vivienda 2002)
Adaptive Capacity	1	Access to communication technologies	Households with mobile phone, landline telephone, internet connection and computer	2002	Instituto Nacional de Estadística de Chile (Censo de Población y Vivienda 2002)
	2	Access to water supply	Households per hectarea without water provision within the house	2002	Instituto Nacional de Estadística de Chile (Censo de Población y Vivienda 2002)
	3	Material Index	Number of houses per hectarea with light materials in external walls	2002	Instituto Nacional de Estadística de Chile (Censo de Población y Vivienda 2002)
	4	Access to medical services	Distance (m) of the centroid of the built up area in the census block to the nearest health care centre	2013	http://www.mapcruzin.com/free-chile-country-city-place-gis-shapefiles.htm
	5	Roads	km/km2 of roads per census tract	2013	http://www.mapcruzin.com/free-chile-country-city-place-gis-shapefiles.htm
	6	Normalized Difference Vegetation Index (NDVI)	NDVI values	2002-2005-2009	Landsat TM / 16.03.2009

### 2.2. Heat vulnerability index

The heat vulnerability index (HVI) was developed based on a Geographic Information System (GIS) spatial analysis. Spatial patterns of heat vulnerability were mapped across the urban fabric using census tracts. A selected set of variables was prepared and statistically analysed. The data cover the 2002–2010 period because of census data availability reasons, as calculations were done by combining remote sensed based indexes with census data (Table 1). To account for spatial concentration patterns, most variables are expressed in relative terms per unit of surface (n/hectare) using the net built up surface within the continuous urban area of each respective census tract. This normalization method prevents the generation of spatial biases induced by very large/small census tracts. Different combinations between exposure, sensitivity and adaptive capacity have been used in the vulnerability literature, by defining vulnerability as multiplicatory or summatory model of certain factors, or introducing the risk as a combination of exposure and sensitivity and then considering adaptive capacity separetly [31,32,33,34]. In this work the HVI was calculated as a function of component impacts (I) and adaptive capacity (A), which in turn are expressed by exposure (E) and sensitivity (S). Within the model, separated quantifications of exposure, sensitivity and adaptive capacity were given to determine variations in vulnerability levels as combinations of individual indexes. This approach can better inform policy making than bulk vulnerability quantifications. Therefore, the HVI value (V) was calculated using a summatory model described by Eq 1:

$$\begin{array}{l} V=f\left(I,A\right)\\ I=\left(E,S\right)\\ or\\ V_{j}=E_{j}+S_{j}-A_{j} \end{array}$$ (1)

where *E*_*j*_ is the exposure level in census tract *j*, *S*_*j*_ is the sensitivity level in census tract *j*, and *A* corresponds to the adaptive capacity of census tract *j*.

#### 2.2.1. Exposure variables

In this study, heat exposure is represented as Land Surface Temperature (LST), which is obtained from the thermal emissivity of land surfaces stored in remote sensing images. This was calculated through standard procedures using the thermal band [35,36,37,38] of three Landsat images taken midday at the end of summer (Table 1). The images were clipped to fit the extension of the continuous urban area. Zonal statistics for each census tract were calculated using ArcGIS 10. Exposure values for each census tract were calculated using the pixel-based LST: the average LST for each census tract plus one standard deviation of temperature for the same census tract, for 2002, 2005 and 2009. This calculation corresponds to a conservative estimation of exposure.

#### 2.2.2. Sensitivity variables

Six variables from the census database were chosen to quantify sensitivity: 1) *elderly population*, 2) *children*, 3) *disabled population*, 4) *family structure*, 5) *education* and 6) *unemployment* (Table 1). The census variable describing the *elderly population* (1) was the total number of people of 65 years of age or older. The *children* (2) variable included the total number of people of five years of age or younger. The *disabled population* (3) corresponds to the sum of six census categories: i) only blindness, ii) only deafness iii) only muteness, iv) paralysis, v) mental illness and v) multiple physical disabilities. For *family structure* (4), marital status was used as a proxy for the family structure based on four census categories to recognise those who live alone: i) single, ii) widowed, iii) divorced and iv) separated. *Education* (5) refers to individuals with lower levels of education based on the last level of formal education completed but including only the four lowest levels: i) never attended, ii) pre-elementary, iii) special differential and iv) basic/elementary. For *unemployment* (6), four categories were considered: i) working for a family without monetary payment, ii) looking for a first job, iii) student and iv) permanently unable to work. According to census criteria, categories for variables 4, 5 and 6 correspond to the head of the household. Data sources and descriptions are shown in Table 1. The total number of cases per category were summed and divided by the net built up area for each respective census tract to obtain the relative spatial variable density. Population data and census tracts were derived from the official Census of 2002 [28], as the most recent census (2012) is not yet available due to technical issues.

#### 2.2.3. Adaptive capacity variables

To measure adaptive capacity levels, six variables were used: 1) *communication*, 2) *no water supply*, 3) *materials*, 4) *medical services*, 5) *roads*, and 6) *the Normalized Difference Vegetation Index (NDVI)* (Table 1). The *communication* (1) variable measures access to communication technologies, and it was calculated based on four categories pertaining to i) Internet, ii) mobile phone, iii) telephone and iv) computer access. To account for co-linearity among these categories (i.e., several households may fall under more than one category), this variable was calculated as the average of all categories plus one standard deviation. The *no water supply* (2) variable accounts for water supply access measured as the number of households without running water. Another relevant variable pertains to building materials, as some materials (e.g., bricks) perform better at night conditions than other materials (e.g., metal panels) due to differences in thermal inertia [39]. The *materials* variable (3) was calculated as the total number of households that use light materials per census tract, based on the following three categories: i) wood lined septum, ii) cement with fibrous materials (a light construction material commonly used in Chile), and iii) waste (tin, cardboard, plastic, etc.). The *medical services* (4) variable measures access to medical services, and it was quantified as the distance from the centroid of a census tract built up area to the nearest health care centre. The closest distance was calculated using a proximity tool based on the centroid of each census tract built up area to avoid biases related to the shapes of census tracts. The *roads* (5) variable was calculated as the paved road density per squared km within a census tract. The *NDVI* (6) was used as a vegetation cover variable. The NDVI provides direct information on the extent and composition of vegetation cover. It was computed as the average NDVI value per census tract plus one standard deviation, to provide a conservative estimation of higher values. NDVI values were obtained using ENVI software and were derived from Landsat imagery (Table 1) using standard procedures. All spatial statistics were calculated using ArcGIS 10 software.

### 2.3. Statistical analyses

The above-described variables do not equally explain heat vulnerability patterns. However, a priori assumptions relating to the relative importance of the original variables in terms of their contributions to vulnerability may produce biases [40]. At the same time, there are likely strong correlations between socioeconomic variables. A principal component analysis (PCA) was conducted to avoid co-linearity issues and to limit the complexity of the variables by reducing the set of original variables to a smaller number of principal components that account for most of the variance in the observed variables. The variables were weighted using the variance weighted approach [41] by aggregating the variance explained by respective components to produce a combined principal component (PC) score (z-score) [40]. The ranking of PCs in order of significance—based on how much data variability they capture—is denoted by *eigenvalues* associated with the vector for each PC [42,43]. The Pearson correlation matrix and Kaiser's recommendations were used for factor retention, where only PCs with *eigenvalues* greater than 1.0 were retained. An orthogonal (Varimax) rotation of *eigenvectors* (factors) was used to improve our interpretations and to maximise the dispersion of loadings across the PCs, producing a set of interpretable factors revealing the simple structure [42,43]. The z-score matrix represents a new group of uncorrelated variables that allow for further mathematical manipulation. The PCs collected for sensitivity and adaptive capacity were transformed into a vector of z-score values, keeping the original structure of the data with 0 values in the centre of the vector and while respecting the weight and signs of the z-scores. For the *n* retained PCs, z-scores were generated for all 277 census tracts, thus ensuring that each one included *n* PC scores. A PCA analysis was conducted using XLSTAT 2015 for Excel. Partial results for exposure, sensitivity and adaptive capacity were normalized to a scale of 0 to 1 using Eq 2.

$$\beta =\left[\frac{x-x_{\text{min}}}{x_{\text{max}}-x_{\text{min}}}\right]$$ (2)

where *β* corresponds to the normalized value, χ is the original value and χ_min_ and χ_max_ are the minimum and maximum values of the dataset, respectively. Normalized values were grouped into five categories using equal intervals to show the spatial distributions at the census tract level. After normalizing the partial scores, the partial vulnerability value was calculated using Eq 1 and then normalized to obtain the final HVI.

#### 2.3.1. Data pre-processing

Scaling and centring pre-processing was conducted to ensure a maximum potential of PCA while avoiding biases produced by large or low variable variance levels. This procedure ensures equal weighting in the data analysis whereby all variables make the same contribution to the model. The sensitivity variables are of the same unit (n/hectares), and thus only scaling and centring were performed. Unit variance scaling was conducted by multiplying variables by the inverse of their standard deviation. Mean centred calculations were performed by determining the average value of each variable and by then subtracting this value from the data. Adaptive capacity variables were measured using different units, and thus standardizing was also performed by subtracting the average and then dividing this result by the standard deviation.

#### 2.3.2. Cluster analisys

To analyse the spatial distribution of indexes a cluster analysis was applied using the Anselin Local I Moran [44]. This statistic of spatial association provides a set of weighted features, identifies statistically significant hot spots, cold spots, and spatial outliers, where spatial clusters of features with attribute values similar in magnitude are identified. The z-scores and p-values represent the statistical significance of the computed index values. Very high or very low (negative) z-scores, associated with very small p-values, are found in the tails of the normal distribution. Small p-values and either a very high or a very low z-score indicates that it is unlikely that the observed spatial pattern reflects the theoretical random pattern represented by the null hypothesis.

## 3. Results

### 3.1. Heat hazard exposure spatial patterns

**Fig 2:**
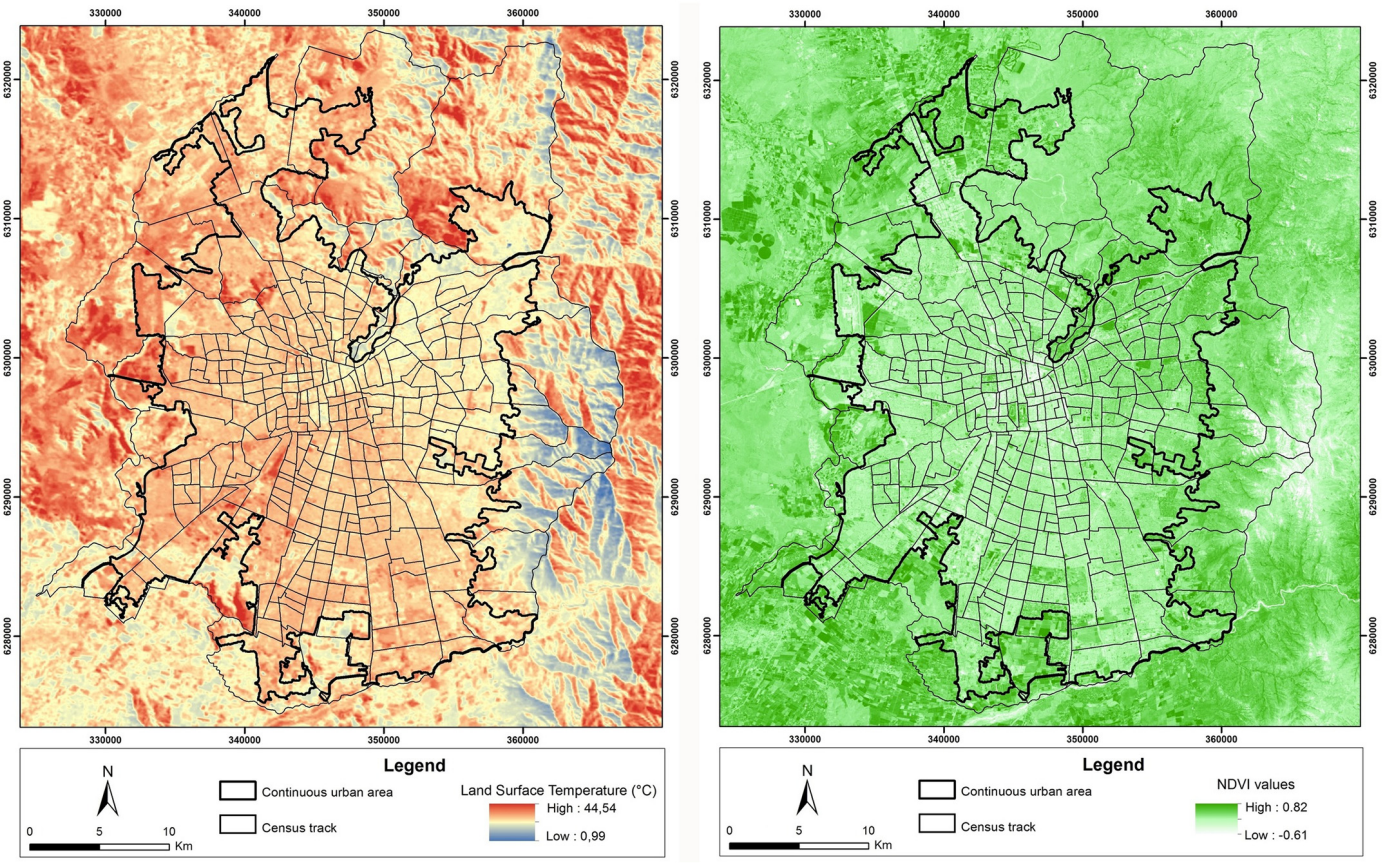
a) Land surface temperature (LST) and b) Normalized diference vegetation index (NDVI) for Santiago. LST and NDVI indexes were calculated based on satellite image taken from NASA Landsat Program, 2009, Landsat TM, scene LT52330832009075COA02, L17, USGS, Sioux Falls, 03/16/2009.

The LST spatial pattern shows considerable differences along the west-east, north and south areas of the city. The highest LST values are found in the western area of the city, where vegetation cover levels are lower. The lowest values are found towards the northeastern region of the urban area. Lower LST values in the central area of the city may be attributable to higher building densities. Large open spaces have a strong cooling effect. In some cases, cooling islands are associated with higher shares of vegetation, i.e., the presence of consolidated green infrastructure, parks or other green areas (Fig 2). In other cases, a less amount of sealed surfaces accounts for lower LST values. It is possible to appreciate the spatial correlation between large open spaces, green areas and lower LST values (Fig 2). A significant correlation was found after comparing 2009 pixels of LST and NDVI (r Pearson = -0.439, p<0.01). The overall LST average for the study period is 31.45 ± -1.43 degrees Celsius. The maximum difference in temperature based on the coolest and warmest places within the continuous urban area is 9.28 degrees Celsius.

The exposure results follow a very clear spatial pattern. Lower values follow the mountains in the eastern area of the city. Higher values are concentrated in the north and west. Exposure depicts the “cone” of high socioeconomic status towards the eastern area of the city, denoting a higher presence of urban green areas that have spatially restricted cooling effects. The average value of exposure was found to be 0.41 (moderate according to the five-point scale used) with a standard deviation of 0.15. The correlation between the distance to the city centre and exposure is not very high but is statistically significant, reaching 0.16, indicating that exposure slightly increases for census tracts away from the city centre. However, this dependency is stronger towards the west than in the east. Correlations between population density and exposure are not statistically significant (-0.08), whereas correlations between the total built-up area and exposure is slightly high (0.21).

### 3.2. Statistical analysis of variables for sensitivity

**Fig 3:**
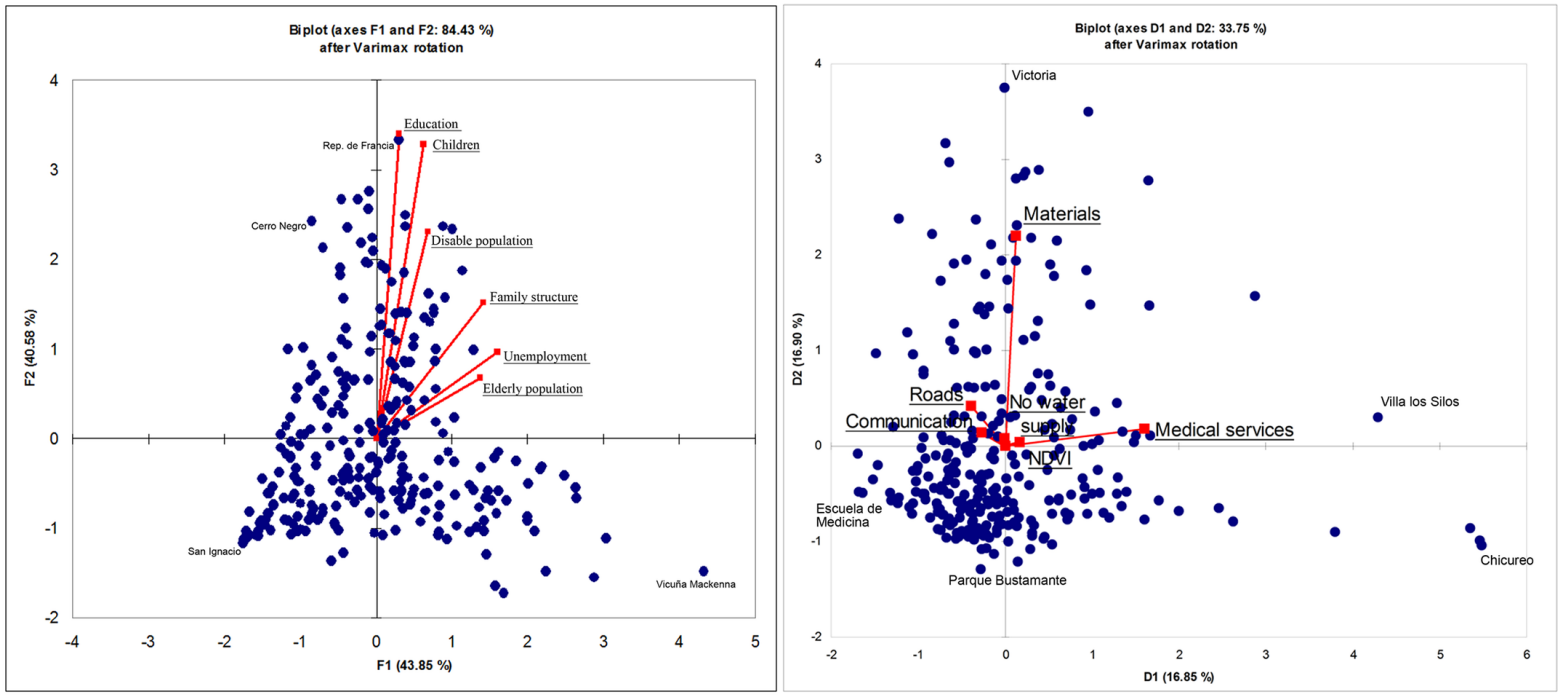
Scatter plots for z-values of sensitivity and adaptive capacity. PC loadings are shown in red.

Bartlett's sphericity test shows a low p-value of < 0.0001. This is significantly lower than the alpha level of 0.05; therefore, the null hypothesis is rejected, i.e., at least one of the correlations between the variables is significantly different from 0. Two PCs were retained. The first PC has a very high *eigenvalue* (4.57) that explains 76.2 percent of the total variance. The second PC is very close to a value of one (0.999), explaining 16.6 percent of the total variance. Most of the data structure was captured in these two components, as both components can explain 92.8 percent of the total variance in the data. The PC structure is simple and clear. The dominating variables in PC1 include the following: *elderly population* (0.82), *family structure* (0.84) and *unemployment* (0.95). Thus, PC1 can be interpreted as “social isolation”. In PC2, dominant variables include the following: *children* (0.91), *disabled population* (0.64) and *education* (0.94). These can be interpreted as “dependency”. The z-score dispersion in addition to the correlations (sign) and magnitudes of the *eigenvectors* (underlying) are shown in Fig 3. Census tracts close to the centre of the plane have average properties whereas those far from one another have asymmetric sensitivity profiles (e.g., Rep. de Francia, which is heavily driven by *education* and San Ignacio, which corresponds to the less sensitive census tract).

The normalized sensitivity presents an average of 0.44 (moderate according to the five-point scale used) and a standard deviation of 0.22. As expected, sensitivity is highly correlated with population density (0.89) and with the total built-up area (0.48). The weight of sensitivity in the HVI is higher than that of exposure (0.67). Correlations between sensitivity and the distance to the centre are low and negative (-0.11), showing that centrality is not relevant for this index. This is consistent with the spatial distribution of sensitivity that shows a cross pattern with an inner ring of lower sensitivity values following certain urban axes towards the north, west and south of the city.

### 3.3. Statistical analysis of variables for adaptive capacity

Three PCs were retained following the Kaiser rule. The first PC has an *eigenvalue* of 2.07, explaining 34.6 percent of the total variance. The second PC has a variance of 1.15, accounting for 19.2 percent of the total variance. The third PC has a variance of 1.04, accounting for 17.4 percent of the total variance. These three PCs retain much of the data structure, accounting for 71.3 percent of the total variance. The rest of the PCs do not fulfil Kaiser’s rule; therefore, they are not considered for further analysis. Following Varimax rotation, eigenvectors of the *medical services* (0.95), *materials* (0.98) and *no water supply* (0.99) variables have very high scores in PCs one, two and three, respectively. *No water supply* and *materials* are the only relevant variables in PCs two and three, respectively. The variables *communication* (-0.15), *roads* (-0.23) and *NDVI* (0.1) present lower loads in the first PC (Table 2). The structure of each PC is neither simple nor straightforward. This is especially the case for the first PC, which is only explained by *medical services*. The z-score dispersion and correlations (sign) and magnitudes of *eigenvectors* (underlying) for adaptive capacity are shown in the scatter plot (c, d) in Fig 3. As in the case of sensitivity, census tracts closer to the centre of the plane have average values whereas those far from one another present asymmetric adaptive profiles (e.g., Victoria, which is strongly driven by *materials* and Villa los Silos, which is driven by *medical services*). Chicureo, Escuela de Medicina and Parque Bustamente are census tracts with higher adaptive capacity values.

**Table 2:** PC loadings for sensitivity (above) and adaptive capacity variables following varimax rotation.

	PC1	PC2	PC3	PC4	PC5	PC6
Elderly population	0.821*	0.188	0.359	0.402	0.006	-0.001
Children	0.376	0.914**	0.099	0.000	-0.026	0.112
Disabled population	0.407	0.644**	0.643	0.079	0.018	0.002
Family structure	0.842*	0.423	0.260	0.070	0.195	0.032
Education	0.179	0.947**	0.235	0.071	0.051	-0.087
Unemployment	0.953*	0.270	0.068	-0.084	-0.085	-0.001
Communication	-0.158*	0.062	0.026	-0.121	0.951	0.229
No water supply	-0.002	0.037	0.997***	-0.041	0.022	-0.040
Materials	0.074	0.984**	0.039	0.019	0.057	0.148
Medical services	0.958*	0.081	-0.003	0.107	-0.155	-0.201
Roads	-0.231*3	0.185	-0.053	-0.107	0.258	0.912
NDVI	0.1*	0.018	-0.043	0.984	-0.110	-0.088

* Statistically significant values for PC1,

** PC2

*** PC3.

The normalized adaptive capacity index is higher for the entire city than exposure and sensitivity levels, with an average value of 0.78 (high according to the five-point scale used) and a standard deviation of 0.15. As this index is inversely related to the HVI, Santiago presents low levels of adaptive capacity overall. Adaptive capacity shows a negative discrete correlation with population density (-0.20) and with the distance to the city centre (0.21), whereas it does not correlate with the total built-up area. Among the three studied indexes, this is the one that most comprehensively (-0.74) explains vulnerability patterns (Table 3). The spatial pattern assumed by adaptive capacity includes a “cone” formed by high socioeconomic status neighbourhoods. This index is higher in census tracts located within the Andean piedmont, with high-income population. However, there is a clear axis of higher adaptive capacity towards the west of the city. Two inner census tracts located in central areas present low adaptive capacity levels: Recoleta and Brasil.

**Table 3:** Pearson correlation matrix (r-values) between main indexes used in the analysis. The built-up density is used as a spatial parameter for comparisons. P-values are significant at 0.01.

	*Density 2002*	*Total bup*	*Total area*	*Exposure*	*Sensitivity*	*Adaptive capacity*	*HVI*	*Distance to centre*
*Density 2002*	1							
*Total bup*	(0.56)	1						
*Total area*	(0.45)	0.74	1					
*Exposure*	(0.08)	0.21	0.12	1				
*Sensitivity*	0.89	(0.48)	(0.36)	(0.20)	1			
*Adaptive capacity*	(0.20)	(0.08)	(0.11)	(0.30)	(0.24)	1		
*HVI*	0.64	(0.19)	(0.13)	0.48	0.67	(0.74)	1	
*Distance to centre*	(0.25)	0.51	0.37	0.16	(0.11)	(0.21)	0.10	1

### 3.4. The HVI for Santiago, Chile

The HVI presents an uneven inner structure, where adaptive capacity (-0.74) and sensitivity (0.67) weigh more than exposure (0.48). As expected, population density is highly correlated with HVI (0.64) but is not strongly dependent on the distance to the city centre (-0.25).

The normalized HVI presents an average of 0.40 (moderate according to the five-point scale used) and a standard deviation of 0.17. Among the 277 census tracts analysed, 41 present high HVI values (Table 4 values of over 0.6). Two of them present very high values: 1) Republica de Francia, despite presenting low exposure levels (0.23), has a higher sensitivity value (1) and an adaptive capacity of zero, 2) Lo Ruiz has the highest exposure value (1) with low sensitivity and adaptive capacity levels as well. These 41 census tracts account for a built-up surface of 6,190 hectares, representing 8 percent of the total continuous urban area.

**Table 4:** List of 41 census tracts with higher HVI values.

	Municipality	Census tract	Exposure	Sensitivity	Adaptive	HVI
1	Huechuraba	República de Francia	0.23	1	0	1
2	Renca	Lo Ruiz	1	0.37	0.23	0.95
3	Santiago	Brasil	0.38	0.74	0.35	0.76
4	Lo Espejo	Las Torres	0.46	0.85	0.56	0.75
5	Cerro Navia	Violeta Parra	0.48	0.82	0.56	0.75
6	Cerro Navia	Victoria	0.5	0.78	0.53	0.75
7	Pudahuel	La Estrella	0.51	0.88	0.66	0.74
8	Cerro Navia	Cerro Navia	0.44	0.73	0.45	0.74
9	Lo Espejo	Población Caro Sur	0.45	0.86	0.6	0.73
10	Cerro Navia	Dalmacia	0.47	0.75	0.52	0.73
11	Renca	Cerro Colorado	0.88	0.5	0.68	0.73
12	San Bernardo	Santa Marta	0.45	0.82	0.6	0.71
13	La Pintana	Pablo de Rokha	0.46	0.78	0.59	0.7
14	El Bosque	Santa Elena	0.49	0.75	0.58	0.7
15	Lo Espejo	Carlos Dittborn	0.41	0.94	0.71	0.69
16	Lo Prado	Blanqueado	0.56	0.95	0.87	0.69
17	Lo Prado	Costa Rica	0.44	0.91	0.72	0.69
18	Pedro Aguirre Cerda	La Feria	0.46	0.83	0.68	0.68
19	Cerro Navia	Janequeo	0.47	0.78	0.63	0.68
20	El Bosque	El Almendro	0.46	0.75	0.62	0.67
21	Pudahuel	Pudahuel	0.85	0.02	0.3	0.66
22	Cerro Navia	Población Roosevelt	0.46	0.8	0.68	0.66
23	Conchalí	Pomaire	0.39	0.65	0.48	0.65
24	El Bosque	Aviadores	0.36	0.77	0.57	0.65
25	Cerrillos	Divino Maestro	0.51	0.61	0.57	0.65
26	Renca	José Miguel Infante	0.41	0.64	0.5	0.65
27	Quilicura	Lo Echevers	0.62	0.42	0.49	0.65
28	Lo Espejo	Población Caro Norte	0.47	0.78	0.72	0.64
29	Huechuraba	Avenida Principal	0.24	0.75	0.47	0.63
30	Pedro Aguirre Cerda	La Victoria	0.46	0.79	0.74	0.63
31	Cerro Navia	José Joaquín Pérez	0.5	0.72	0.7	0.63
32	San Bernardo	Cerro Negro	0.6	0.68	0.76	0.63
33	San Ramón	La Bandera	0.39	0.66	0.55	0.62
34	Lo Espejo	Lo Espejo	0.51	0.62	0.64	0.62
35	Lo Espejo	Quiriquina	0.47	0.75	0.73	0.62
36	Pudahuel	Embalse Lo Prado	0.99	0.24	0.73	0.62
37	La Granja	San Gregorio Oriente	0.39	0.65	0.57	0.61
38	Pedro Aguirre Cerda	Lo Valledor Sur	0.47	0.64	0.64	0.61
39	Estación central	Hermanos Carrera	0.51	0.57	0.61	0.61
40	Cerro Navia	El Montijo	0.72	0.44	0.69	0.61
41	El Bosque	Los Cóndores	0.38	0.68	0.6	0.6

Partial values of exposure, sensitivity and adaptive capacity are shown to determine their weight for the overall HVI.

**Fig 4:**
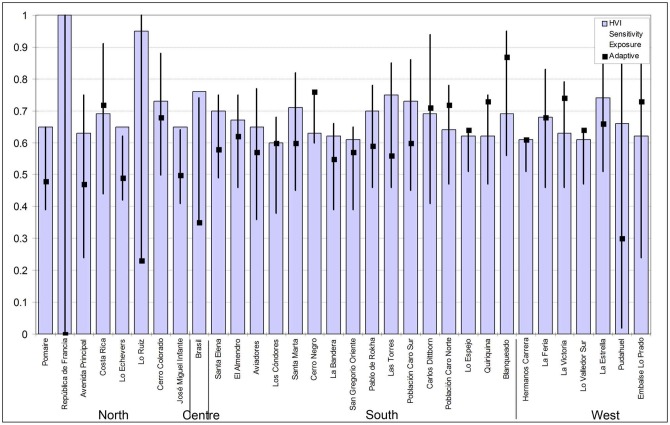
Normalized values for HVI, exposure, sensitivity and adaptive capacity for the 41 census tracts with higher values HVI. HVI increases towards the northern end of the city.

**Fig 5:**
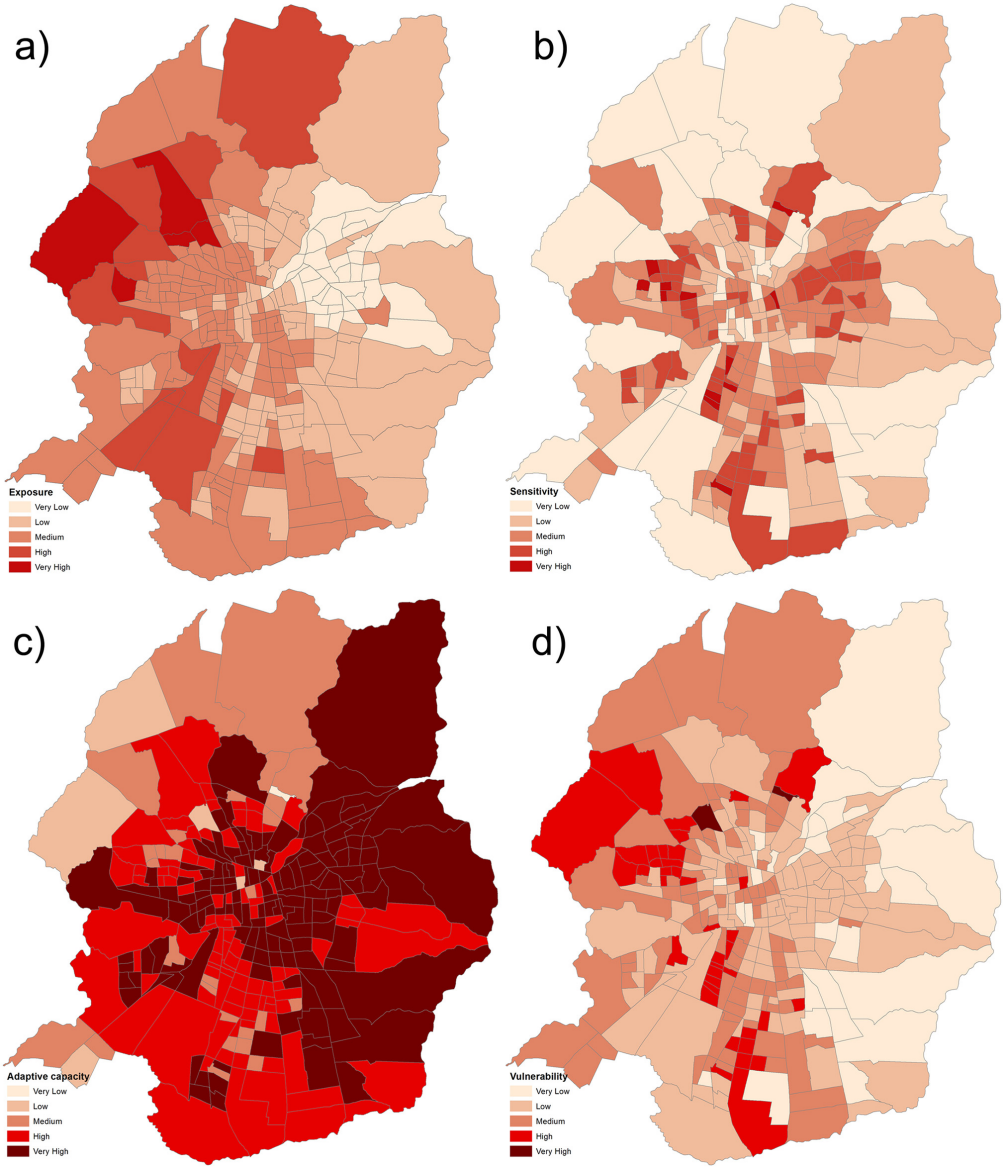
Results for a) exposure, b) sensitivity, c) adaptive capacity and d) the heat vulnerability index at the census tract level for Santiago.

The spatial structure of the HVI imprints the negative value of adaptive capacity (the index of higher weight). Lower HVI values were found in census tracts located in the eastern area of the city and in inner city areas with values ranging from 0.07 to 0.19 (Figs 4 and 5d).

#### 3.4.1. Clusters of heat vulnerability

**Fig 6:**
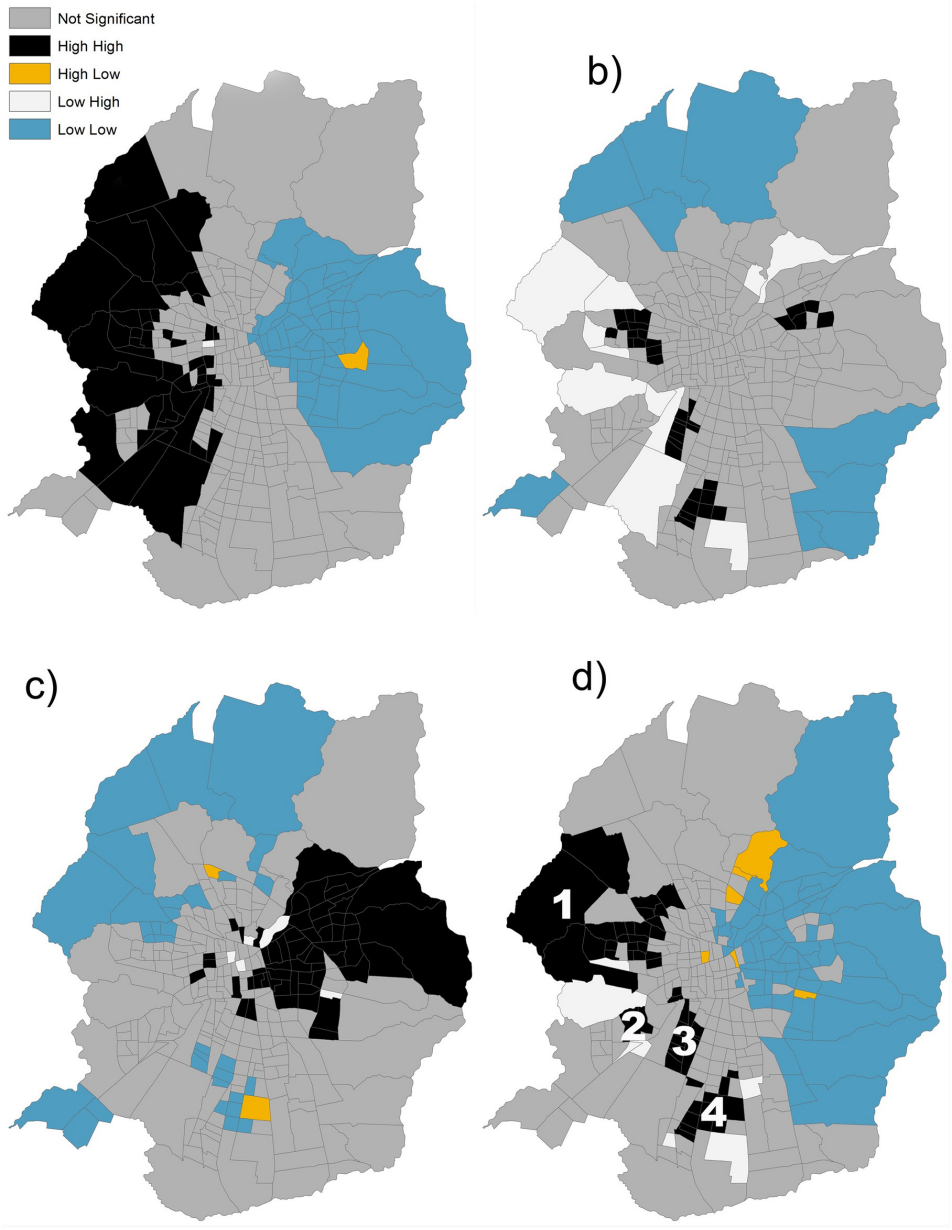
Cluster analysis. Anselin I Moran for exposure a), sensitivity b), adaptive capacity c) and HVI d). The four clusters of high HVI values are indicated in caption d) with the number 1, 2, 3 and 4. High High for a statistically significant (0.05 level) cluster of positive high values (z-scores) and Low Low for a statistically significant (0.05 level) cluster of positive low values. Statistically significant spatial outliers (0.05 level) are indicated by negative z-scores: High Low if the feature has a high value and is surrounded by features with low values and Low High if the feature has a low value and is surrounded by features with high values.

Clusters of high values for the calculated indexes shown an opposite location, where higer values for exposure and sensitivity are located towards the west of the city while higher values for adaptive capacity towards the east of the city, in a spatial mirror east-west. This pattern imprints clearly the spatial distribution of socio-economic classes within the city, confirming other similar results [14]. This unveven combination of partial indexes produces a spatial concentration of high HVI values in the west and southern areas of Santiago. There are four well defined clusters of high HVI values (Fig 6) involving 52 census tracts (Table 5). Cluster 1 is the largest one, with 24 census tracts and accounting for a surface of 4,792 hectares. Cluster 2 is the smallest, only with 2 census tracts and a surface of 586 hectares. Cluster 3 and 4 are similar in extension, including 15 census tracts and 1,482 hectares and 11 census tracts and 1,549 hectares respectively. The correspondence between population density and heat vulnerability is also confirmed: density is higher than the city’s average density (85.2 inhabitants per hectare is the net population density) in 17 of the 24 census tracts of cluster one, in all census tracts in cluster 2, in 14 over 15 census tracts in cluster 3 and 10 over 11 census tracts in cluster 4.

**Table 5:** List of 52 census tracts contained in the four indicated clusters.

Cluster	Municipality	Census tract	Density 2002	Exposure	Sensitivity	Adaptive	HVI
1	Quinta Normal	Catamarca	115.6	0.41	0.53	0.65	0.51
	Quinta Normal	Frontera	133.7	0.41	0.63	0.76	0.51
	Lo Prado	Lo Prado	149.4	0.45	0.63	0.74	0.54
	Lo Prado	Blanqueado	181.6	0.56	0.95	0.87	0.69
	Lo Prado	Territorio Ant rtico	160.8	0.41	0.71	0.76	0.55
	Lo Prado	Costa Rica	186.2	0.44	0.91	0.72	0.69
	Pudahuel	Embalse Lo Prado	46.8	0.99	0.24	0.73	0.62
	Pudahuel	Barrancas	123.9	0.48	0.55	0.71	0.53
	Pudahuel	La Estrella	188.9	0.51	0.88	0.66	0.74
	Pudahuel	Santa Corina	35.8*	0.67	0.48	0.82	0.53
	Pudahuel	Pudahuel	0.5*	0.85	0.02	0.3	0.66
	Cerro Navia	Cerro Navia	135.1	0.44	0.73	0.45	0.74
	Cerro Navia	Janequeo	166.9	0.47	0.78	0.63	0.68
	Cerro Navia	Jos, Joaqu¡n P,rez	154.3	0.5	0.72	0.7	0.63
	Cerro Navia	Poblacion Roosevelt	181.3	0.46	0.8	0.68	0.66
	Cerro Navia	Dalmacia	157.3	0.47	0.75	0.52	0.73
	Cerro Navia	El Montijo	47.2*	0.72	0.44	0.69	0.61
	Cerro Navia	Violeta Parra	165.4	0.48	0.82	0.56	0.75
	Cerro Navia	Victoria	166.1	0.5	0.78	0.53	0.75
	Renca	Renca	97.1*	0.45	0.48	0.67	0.5
	Renca	Lo Ruiz	51.4*	1	0.37	0.23	0.95
	Renca	Jos, Miguel Infante	130.8	0.41	0.64	0.5	0.65
	Renca	Cerro Colorado	47.8*	0.88	0.5	0.68	0.73
	Quilicura	Lo Echevers	25.8*	0.62	0.42	0.49	0.65
2	Cerrillos	Divino Maestro	119.0	0.51	0.61	0.57	0.65
	Maipu	Los Libertadores	123.9	0.5	0.65	0.82	0.53
3	El Bosque	Lagos de Chile	123.4	0.36	0.56	0.5	0.58
	Pedro Aguirre Cerda	Miguel D vila	132.9	0.4	0.57	0.68	0.51
	Pedro Aguirre Cerda	La Feria	187.8	0.46	0.83	0.68	0.68
	Pedro Aguirre Cerda	Lo Valledor Norte	96.3	0.53	0.45	0.69	0.51
	Pedro Aguirre Cerda	Lo Valledor Sur	144.3	0.47	0.64	0.64	0.61
	Pedro Aguirre Cerda	Navidad	153.7	0.41	0.6	0.72	0.51
	Lo Espejo	Lo Espejo	127.1	0.51	0.62	0.64	0.62
	Lo Espejo	Las Torres	178.6	0.46	0.85	0.56	0.75
	Lo Espejo	Poblacion Caro Sur	190.2	0.45	0.86	0.6	0.73
	Lo Espejo	Poblacion Caro Norte	165.6	0.47	0.78	0.72	0.64
	Lo Espejo	Quiriquina	168.6	0.47	0.75	0.73	0.62
	Lo Espejo	Carlos Dittborn	203.9	0.41	0.94	0.71	0.69
	Lo Espejo	Clara Estrella	73.9*	0.61	0.4	0.7	0.52
	Estacion central	Hermanos Carrera	133.3	0.51	0.57	0.61	0.61
	Estacion central	Nogales	163.2	0.49	0.74	0.8	0.59
4	La Pintana	Pablo de Rokha	166.4	0.46	0.78	0.59	0.7
	La Pintana	Antumapu	46.3*	0.67	0.54	0.88	0.53
	San Ramon	Paraguay	163.6	0.41	0.7	0.68	0.59
	San Ramon	La Bandera	143.9	0.39	0.66	0.55	0.62
	El Bosque	Aviadores	160.7	0.36	0.77	0.57	0.65
	El Bosque	Santa Elena	155.0	0.49	0.75	0.58	0.7
	El Bosque	El Almendro	142.1	0.46	0.75	0.62	0.67
	El Bosque	Leon XIII	138.5	0.42	0.7	0.76	0.55
	El Bosque	Los Condores	148.1	0.38	0.68	0.6	0.6
	San Bernardo	Calderon de La Barca	166.4	0.48	0.76	0.86	0.56
	San Bernardo	Santa Marta	159.6	0.45	0.82	0.6	0.71

Respective municipalities to which they belong to are indicated. Population density for the year 2002 it is indicated as well. Density values lower than the average net population density are shown with *.

In fact average population density is very high in the four clusters: 118.7, 121.5, 149.5 and 144.6 respectively. Minimum HVI values are extremely high in all clusters: 0.5 in cluster 1, 0.53 in cluster 2, 0.51 in cluster 3 and 0.53 in cluster 4 respectively. The highest HVI mean value was to be found in cluster 1 (0.65).

## 4. Discussion

In this section, five main issues will be discussed. They refer to the exploration of results and to methodological aspects: 1) sensitivity analysis of the heat vulnerability clusters; 2) PC structure for sensitivity and adaptive capacity and 3) index spatial structures; 4) strengths and shortcomings of the selected variables; 5) study area delineation; and 6) transferability of results.

### 4.1. Exploration of results

Clusters of high heat vulnerability values involve 15 municipalities (Table 5). In some cases like the municipalities of Lo Espejo and Cerro Navia, the total municipality’s surface presents higher HVI values. For such municipalities a clear consideration of urban planning measures towards urban heat should be a priority. In general high HVI values in these clusters are the result of higher exposure and lower adaptive capacity (Table 5).

#### 4.1.1. Sensitivity analysis of the heat vulnerability clusters

To explore and understand the heat vulnerability clusters the data structure of variables in those specific census tracts in comparison with the HVI values was analysed. For this purpose a correlation matrix was performed between HVI and the all the original variables used in the PCA calculations (Table 6). Pearson correlation is statistically significant for *children* (0.38) and *education* (0.39), both variables corresponding to the sensitivity index. The higher correlation (r-values) were found between HVI and *no water supply* (0.44) and *materials* (0.46), respectively, both variables for adaptive capacity. To ascertain the eventual impact of *no water supply* and *materials* in the performance of the HVI we ran a sensitivity analysis. Looking at the PCA factor loadings (Table 2) we found that those two variables correspond to the second and the third components for adaptive capacity. They explain 19.2% and 17.4% of the total variability, both together explaining 36.6% of the total variability of the adaptive capacity index. In adition they are the only relevant factors in those two components. For that reason the sensitivity analysis was performed over PC2 and PC3 for adaptive capacity.

**Table 6:** Correlation matrix (r-values) between HVI and the original variables used in the PCA.

Variable	Correlation with HVI	
LST	0.30	Exposure
Elderly population	-0.03	Sensitivity
Children	0.38	
Disable population	0.20	
Family structure	0.14	
Education	0.39	
Unemployment	0.11	
Communication	0.06	Adaptive
No water supply	**0.44** *	
Materials	**0.46** *	
Medical services	-0.09	
Roads	-0.07	
NDVI	-0.05	

* indicates statistically significant values.

The original rotated values in components 2 and 3 in the 52 census tracts of high HVI values (4 clusters) were manipulated in two rounds. The original rotated values were substitued by 1) the average rotated value of the respective component in the first round and 2) the minimum rotated value in the second round. The idea is to estimate how much of the HVI will improve if those two components would have the average and the mimimum value for the city. The average rotated value represents a feasible improved scenario, where *no water supply* and *materials* in those census tracts are improved up to the city’s average. The minimum rotated value represents the ideal scenario, were *no water supply* and *materials* are improved to the maximum city’s value. With this technique the original variability of the data in terms of range and frequency distribution is kept. The impact of this matematical operation in the adaptive capacity values of census tracts located out of the analysed clusters was also calculated. The absolute change between the original adaptive capacity value and the new adaptive capacity value after the substitution was to be found -0.104 for the first round and -0.075 for the second round.

**Fig 7:**
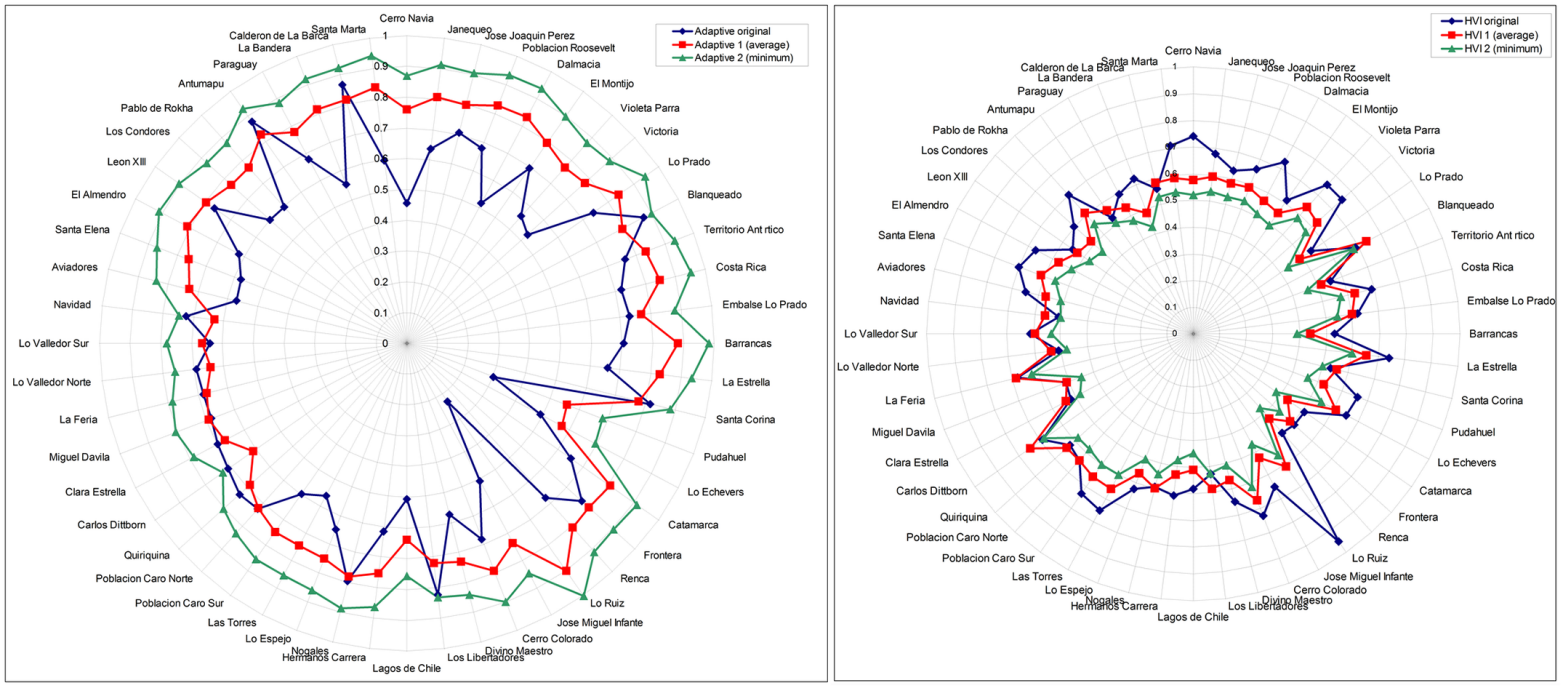
Spider plot for the sensitivity analysis for adaptive capacity (left) and HVI (right) for the 52 census tracts existing in the four heat vulnerability clusters.

The sensibility analysis shows that important improvements in terms of adaptive capacity and heat vulnerability can be done by improving the water supply and material conditions of the census trats located in the heat clusters. The mean adaptive capacity for the 52 census tracts increased from 0.65 to 0.76 in the adaptive 1 (average) and up to 0.87 in the adaptive 2 (minimum) (Fig 7). The higher increase in adaptive capacity was found in Lo Ruiz which went from an original adaptive capacity of 0.23, up to 0.9 (average) and 1 (minimum) and for HVI went from original HVI of 0.95, up to 0.60 (average) and 0.55 (minimum). The average increase in adaptive capacity for all 53 census tracts is 0.11 (average) and 0.22 (minimum), and for HVI is 0.06 (average) and 0.11 (minimum). Partial values for every cluster are shown in Table 7.

**Table 7:** Main statistics for partial indexes and HVI calculated for four clusters.

*Cluster 1*	*Density 2002*	*Total hectares*	*Exposure*	*Sensitivity*	*Adaptive*	*Adaptive 1*	*Adaptive 2*	*HVI*	*HVI original*	*HVI 1*	*HVI 2*
Mean	118.73	199.67	0.57	0.62	0.63	0.79	0.89	0.65	0.65	0.57	0.51
Standard Error	12.04	44.06	0.04	0.05	0.03	0.02	0.01	0.02	0.022	0.02	0.02
Median	134.39	113.55	0.48	0.64	0.68	0.80	0.90	0.66	0.66	0.58	0.52
Standard Deviation	58.98	215.86	0.19	0.22	0.15	0.08	0.07	0.11	0.11	0.08	0.08
Sample Variance	3,479.06	46,593.93	0.03	0.05	0.02	0.01	0.01	0.01	0.01	0.01	0.01
Range	188.38	811.47	0.59	0.93	0.64	0.35	0.32	0.45	0.45	0.31	0.31
Minimum	0.47	47.33	0.41	0.02	0.23	0.56	0.68	0.50	0.50	0.435	0.37
Maximum	188.85	858.80	1.00	0.95	0.87	0.90	1.00	0.95	0.95	0.73	0.68
Count	24	24	24	24	24	24.00	24.00	24	24	24	24
Confidence Level(95.0%)	24.91	91.15	0.08	0.09	0.07	0.03	0.03	0.04	0.04	0.03	0.03
*Cluster 2*											
Mean	121.45	293.00	0.51	0.63	0.70	0.72	0.83	0.59	0.59	0.58	0.52
Standard Error	2.48	38.30	0.01	0.02	0.13	-	-	0.06	0.06	0.011	0.011
Median	121.45	293.00	0.51	0.63	0.70	0.72	0.83	0.59	0.59	0.58	0.52
Standard Deviation	3.51	54.16	0.01	0.03	0.18	-	-	0.08	0.08	0.02	0.02
Sample Variance	12.35	2,933.78	0.00	0.00	0.03	-	-	0.01	0.01	0.00	0.02
Range	4.97	76.60	0.01	0.04	0.25	-	-	0.12	0.12	0.02	0.02
Minimum	118.96	254.70	0.50	0.61	0.57	0.72	0.83	0.53	0.53	0.56	0.51
Maximum	123.93	331.30	0.51	0.65	0.82	0.72	0.83	0.65	0.65	0.59	0.53
Count	2	2	2	2	2	1.00	1.00	2	2	2	2
Confidence Level(95.0%)	31.57	486.65	0.06	0.25	1.59	-	-	0.76	0.76	0.14	0.13
*Cluster 3*											
Mean	149.51	98.81	0.47	0.68	0.67	0.69	0.81	0.61	0.61	0.60	0.54
Standard Error	9.27	12.83	0.02	0.04	0.02	0.01	0.01	0.02	0.02	0.02	0.02
Median	153.68	97.53	0.47	0.64	0.68	0.69	0.80	0.61	0.61	0.59	0.53
Standard Deviation	35.92	49.70	0.06	0.16	0.08	0.05	0.05	0.08	0.08	0.07	0.07
Sample Variance	1,290.28	2,470.33	0.00	0.03	0.01	0.00	0.00	0.01	0.01	0.01	0.01
Range	130.06	184.27	0.25	0.54	0.30	0.17	0.16	0.24	0.24	0.24	0.24
Minimum	73.88	39.03	0.36	0.40	0.50	0.61	0.73	0.51	0.51	0.51	0.48
Maximum	203.94	223.30	0.61	0.94	0.80	0.78	0.89	0.75	0.75	0.75	0.68
Count	15	15	15	15	15	15.00	15.00	15	15	15	15
Confidence Level(95.0%)	19.89	27.52	0.03	0.09	0.04	0.03	0.03	0.04	0.04	0.04	0.04
*Cluster 4*											
Mean	144.60	140.83	0.45	0.72	0.66	0.79	0.90	0.63	0.62	0.56	0.50
Standard Error	10.28	14.25	0.03	0.02	0.04	0.01	0.01	0.02	0.02	0.01	0.01
Median	155.01	140.10	0.45	0.75	0.60	0.80	0.90	0.62	0.62	0.57	0.51
Standard Deviation	34.08	47.26	0.08	0.08	0.12	0.03	0.03	0.06	0.06	0.04	0.04
Sample Variance	1,161.36	2,233.25	0.01	0.01	0.01	0.00	0.00	0.00	0.00	0.00	0.00
Range	120.03	178.87	0.31	0.28	0.33	0.11	0.10	0.18	0.18	0.13	0.12
Minimum	46.34	69.33	0.36	0.54	0.55	0.73	0.84	0.53	0.53	0.48	0.43
Maximum	166.37	248.20	0.67	0.82	0.88	0.84	0.94	0.71	0.71	0.61	0.56
Count	11	11	11	11	11	11.00	11.00	11	11	11	11
Confidence Level(95.0%)	22.89	31.75	0.06	0.05	0.08	0.02	0.02	0.04	0.04	0.03	0.03

Values for sensitivity analysis perfomed over adaptive capacite are also shown, as well as new values for adjusted HVI. Adaptive 1 and HVI 1 correspond to the first round (average). Adaptive 2 and HVI 2 to the second round (minimum).

The two analysed variables (*no water supply* and *materials)* they explain in a large proportion the hight levels of heat vulnerability in the heat clusters. Those variables are as well clear indicators of informal urban development, which remains high in Santiago [13]. There is a direct relationship between heat vulnerability and informal urban development which can be further explored.

#### 4.1.2. PC structure for sensitivity and adaptive capacity indexes

The six complex variables used for sensitivity index were successfully reduced to two PCs: social isolation (PC1) and dependency (PC2). Social isolation groups include the *elderly population*, *family structure* and *unemployment* variables. When combined, these variables produce a weak social network that largely includes those who experience difficulties in obtaining support during times of need. Dependency refers to those who depend on someone else due to age (*children*) or social conditions (e.g., students in the case of *education* or *disabled population)*. Both PCs can assist in the monitoring and management of sensitive groups in regards to heat hazards. On the other hand, the adaptive capacity results show more complex behaviours. The resulting PCs did not clearly group the variables in a way that is easy to interpret. Variables such as *communication*, *NDVI* and *roads*, which are in principle central to determining social responses to heat hazards, were not found to be relevant in explaining variability levels between the components examined (PC1, PC2 and PC3). There are several reasons for this weak statistical trend. First, the complexities of the phenomenon and of corresponding social responses complicate the selection of the best variables at the correct scale. In this way, exposure and sensitivity are straightforward indexes. Second, one must consider the nature of the data used, as institutional responses may be expressed at different spatial scales (in this case, municipalities rather than census tracts).

#### 4.1.3. Spatial structure of the indexes

The results of the distance to the centre analyses (Table 3) were not clear enough to denote specific spatial dependency or centrality effects. Overall, the distance to the centre has a minor effect on sensitivity and exposure and a slightly greater effect on adaptive capacity. The overall effect on HVI is minor (0.1).

Several studies use population density as variable for sensitivity [40,45]. In this study, population density was excluded from the initial collection of variables, as it corresponds to a structural feature of urban tissues that follows specific spatial structure that in turn affects the core composition of the urban socioeconomic dimension. It is expected that higher population densities will lead to higher levels of sensitivity in a very direct manner. In contrast, one of the purposes of this study was to assess specific socio-economic features that govern spatial behaviours of heat sensitivity and vulnerability. Such facts are shown by specific correlation degrees of population density with HVI (0.64) and sensitivity indexes (0.89) (Table 3).

We found different spatial patterns for exposure, sensitivity and adaptive capacity and weak correlations between them. This can be explained by the lack of specific adaptive actions focused on heat hazards without special capacities designed for the most sensitive or exposed areas. Overall, highly exposed areas do not correspond directly with the most sensitive or least prepared areas. However, the HVI shows a clear spatial asymmetry where the highest vulnerability values are spread across several parts of the city, especially in suburban districts towards the north, south and west, particularly where the low-income population is located. Despite minor differences, other studies confirm these spatial patterns, with higher LST and UHI intensity levels found in the neighborhoods with concentration of vulnerable groups of population, less public infrastructure (e.g. for traffic, health, commerce purposes) and less availability of urban vegetation (the coolest regions) [26, 46, 27].

### 4.2. Methodological discussion

#### 4.2.1. Strengths and shortcomings of the selected variables

We selected certain exposure, sensitivity and adaptive capacity variables to address most elements that can explain heat vulnerability based on the scientific literature. However, our variable selection method was limited by the quality and spatial resolution of available data. Spatially explicit, high quality information remains a key constraint on research in the Latin American context [47]. For exposure, *LST* was used as a variable obtained from available LANDSAT satellite imagery. Several studies have used air temperature as an indicator of exposure (see [48] for a review). Other studies have taken a different approach by examining Physiologic Equivalent Temperature (PET), which includes additional variables such as air humidity and wind velocity. PETs can be higher than air temperatures, and especially in the summer. This in turn helps one better understand thermal effects on individuals [49]. Both are strong descriptors of thermal (dis)comfort, but PET data collection involves extensive fieldwork, making it more expensive and time intensive. By contrast, we perform a vulnerability analysis based on available data while recognizing the strong correlation between *LST* and air temperature [50,51,52,53,27]. This methodological approach ensures the transferability of our results to similar contexts in a simple and straightforward manner that supports policy making.

Some studies show that social stratification across a city matches the spatial distribution of heat exposure [54]. In examining such correlations, spatial data related to heat exposure can be derived from LSTs listed in satellite images. The assumption here is that surface temperature can be representative of air temperature [40,55,56].

Most studies that have quantified sensitivity levels have focused on age groups that are more sensitive to heat, i.e., the elderly and children [57,58,59]. Such information is broadly available in census data and is possible to map with relatively little effort. Other complementary variables (especially in terms of household characteristics such as knowledge and preparation) that support warning or recovery information are more difficult to quantify. For this reason, we use *education* as a proxy variable to depict this type of sensitivity. A similar case is found for *family structure* and *unemployment*, for which the direct link with income, which is a more explicit variable, might vary.

Understanding adaptive capacity in a meaningful way is crucial to assessing vulnerability, though this is difficult to accomplish due to a lack of specific data available and due to high costs associated with obtaining them. Alternatively, generic information is often used (e.g., GDP per capita, literacy levels, social organization, etc. [60]. However, the appropriateness of using these data remains uncertain [5]. The adaptive capacity index was quantified based on household and territorial capacities. However, the selected variables performed worse than they did for sensitivity. Variables such as *communication*, *NDVI* and *roads* only marginally explained the adaptive capacity variability. In fact, responses to heat hazards are complex and depend heavily on existing social institutions, thus producing a scale conflict with the spatial unit used (the census tract, which lacks such institutional representation). Other similar studies that have used more aggregated spatial units (municipalities) have found stronger PC performance for adaptive capacity based on the same variables [8]. According to the existing literature [61,62] social capital is the main resource that prevents and responds to heat hazards. Despite the weak performance of the PC, the spatial pattern it depicts is clear and consistent, demonstrating that adaptive capacity is scale dependent as related to institutional dimensions of a city.

#### 4.2.2. Delineation of the study area

The spatial delineation of urban areas studied and their separation from rural counterparts are fundamental aspects of research studies that address heat effects of cities. Many studies have used administrative boundaries or census spatial units, as they allow matching socioeconomic data in performing spatial analyses. However, such administrative boundaries do not necessarily follow physical extensions of cities, producing spatial biases, especially in peripheral districts. To avoid this methodological shortcoming, in this study, spatial indexes were strictly calculated from continuous urban fabrics within census tracts. For this purpose, an ad hoc spatial delimitation for joining socioeconomic data at the intra-city level obtained from census data was performed.

#### 4.2.3. Transferability of the results

The methodology employed was developed to analyse Santiago de Chile according to the availability of spatial data. The index may be applied with slight adjustments to other cities in South America that could be affected by heat stress. Some interesting cases to study in the future are the megacities like Lima (Peru), Buenos Aires (Argentina), Sao Paulo and Rio de Janeiro (Brasil), wich are located on the coast [63,64]. For such analyses, sensitivity and adaptive capacity indexes can be improved by adding context-specific variables that may vary case by case. For instance, when a coastal city is studied, adaptive capacities should consider variables related to the existence of sea thermal inertia and breeze, etc. Urban form has strong effecs on wind circulation patterns, which in many cases affect diurnal cooling effects of shadow generation in dense urbanized zones [65]. Another relevant aspect in the South American continent is the context specific connection between heat vulnerability and informal urban development [3] which can be further explored.

The main restriction related to the transferability of this methodology pertains to the availability of spatial data. The political division of cities varies considerably across the continent, being completely different in terms of nomenclature, size, etc. from country to country. The trade-off between the smallest spatial division and the robustness of the statistical data must be carefully assessed and discussed in each case to identify an optimal fit between spatial resolution and data availability.

Finally, it has to be noticed that this work focused on the daytime temperatures, due to the higher probability to reach the absolute maximum temperature at this time. However, considering that the UHI effect is generally more intensive at night, it might be interesting to study the temporal variation of the proposed index between day and night. Thus, utilizing night time temperatures and expanding the temporal scope of the study is a promising avenue for future research.

## 5. Conclusion

The aim of this research was to analyse heat hazard vulnerability levels in Santiago, Chile. For this purpose, a heat vulnerability index (HVI) was proposed. This spatially explicit index is composed of spatially explicit indexes of exposure, sensitivity and adaptive capacity following the IPCC vulnerability method. The HVI allows one to explore patterns in spatial distributions by identifying clusters of areas with high and low levels of vulnerability (Fig 6) to determine driven factors of hot spots of vulnerability at scales smaller than the municipality level. Variables greatly affecting the performance of the HVI were tested in a sensitivity analysis to show the strong impact of specific improvements in terms of water supply and housing materials.

Heat vulnerability in Santiago shows high levels of asymmetry in terms of spatial distribution, primarily affecting the poorest urban areas and with increasing divergence as a result of current urban development trends. Now is the right time to respond through appropriate urban planning adaptation measures to better prepare the most vulnerable areas which were identified in four clusters and 52 census tracts. Furthermore, specific measures for those heat vulnerability clusters must include improvements in water supply and in housing materials, rather than more green areas or other standard CC adaptation measures.

This work provides evidence of the unequal distribution of adaptive capacity in Santiago. Moreover, this unequal distribution is exacerbating the effects of heat vulnerability: while higher temperatures occur in the northwestern areas, adaptive capacities are most robust in the southeastern areas, where they will be less necessary. Spatial distribution patterns of the sensitivity index are less clear, presenting higher levels of sensitivity in some central and southern zones of Santiago.

The spatial configuration of exposure, sensitivity, adaptive capacity and heat vulnerability indexes can be used to inform urban planning and management and as a platform for public discussions. Location of new large infrastructures such as urban parks, large commercial centres or parking lots, should take into account their impact in terms of HVI values. Results can be used to conduct open and participatory discussions to improve urban planning and land policy regulations in coping with heat stress. Infrastructural, institutional, social and financial adaptation strategies must be combined to limit heat hazards. Adaptation strategies that depend on public investment should be mostly focused on identified zones characterized by lower levels of adaptive capacity.

## Supporting Information

S1 Fig XLS file containing the PCA calculations for sensitivity. (XLS)

S2 Fig XLS file containing the sensitivity analysis for adaptive capacity and HVI. (XLS)

S3 Fig XLS file containing the PCA calculations for adaptive capacity. (XLS)
